# Comparing methods of performing geographically targeted rural health surveillance

**DOI:** 10.1186/s12982-020-00090-0

**Published:** 2020-11-23

**Authors:** David C. Lee, Nancy A. McGraw, Kelly M. Doran, Amanda K. Mengotto, Sara L. Wiener, Andrew J. Vinson, Lorna E. Thorpe

**Affiliations:** 1 Ronald O. Perelman Department of Emergency Medicine, NYU School of Medicine, 462 First Avenue, Room A345, New York, NY 10016 USA; 2 Department of Population Health, NYU School of Medicine, New York, NY USA; 3 Sullivan County Public Health Services, Liberty, NY USA

**Keywords:** Health surveillance, Chronic disease, Geographic information systems, Survey methodology, Rural health

## Abstract

**Background:**

Worsening socioeconomic conditions in rural America have been fueling increases in chronic disease and poor health. The goal of this study was to identify cost-effective methods of deploying geographically targeted health surveys in rural areas, which often have limited resources. These health surveys were administered in New York’s rural Sullivan County, which has some of the poorest health outcomes in the entire state.

**Methods:**

Comparisons were made for response rates, estimated costs, respondent demographics, and prevalence estimates of a brief health survey delivered by mail and phone using address-based sampling, and in-person using convenience sampling at a sub-county level in New York’s rural Sullivan County during 2017.

**Results:**

Overall response rates were 27.0% by mail, 8.2% by phone, and 71.4% for convenience in-person surveys. Costs to perform phone surveys were substantially higher than mailed or convenience in-person surveys. All modalities had lower proportions of Hispanic respondents compared to Census estimates. Unadjusted and age-adjusted prevalence estimates were similar between mailed and in-person surveys, but not for phone surveys.

**Conclusions:**

These findings are consistent with declining response rates of phone surveys, which obtained an inadequate sample of rural residents. Though in-person surveys had higher response rates, convenience sampling failed to obtain a geographically distributed sample of rural residents. Of modalities tested, mailed surveys provided the best opportunity to perform geographically targeted rural health surveillance.

## Introduction

Though 60 million Americans live in rural areas, their health issues are often understudied [1]. Existing surveys confirm that rural health burdens are substantial, but programs to address these needs are underfunded and few are guided by local surveillance [2]. To compound these problems, worsening socioeconomic conditions in rural America have been fueling increases in chronic disease, drug and alcohol abuse, and poor health [3, 4]. However, resources available to track rural trends with sufficient geographical detail are severely lacking [5, 6]. Unlike many urban public health departments that have larger budgets, rural public health departments often have a smaller workforce and more limited funding, which make it hard to prioritize epidemiologic surveillance [7].

Though rural areas of the country may not have the same demographic and socioeconomic diversity as urban regions, there are other factors such as accessibility to resources (e.g., food sources, healthcare) that vary among rural residents and can lead to within-county disparities in health outcomes [8]. There is strong evidence from studies in urban areas that disease prevalence varies significantly at a sub-county level [9]. In order to perform these studies in rural areas, it would be necessary to obtain more geographically precise estimates of disease prevalence.

Nationally, the Centers for Disease Control and Prevention (CDC) administers telephone-based health surveys by county using the Behavioral Risk Factor Surveillance System (BRFSS) [10]. However, in a rural area like New York’s Sullivan County, these annual assessments capture responses for only 40 to 50 of its nearly 60,000 adult residents. This small sample size makes it impossible to provide a geographically detailed assessment of how disease burden varies at a sub-county level [9]. Better health surveillance is needed in places like Sullivan County, as it is ranked second to last in health outcomes and has had the highest rate of premature death in New York State [11].

Compared to urban areas, the following conditions make rural health surveillance more challenging [12]. In Sullivan County, there are two area codes: 845 and 607, which each span across multiple counties. Therefore, traditional methods like random-digit-dialing would result in most calls being made to residents in the wrong county. Furthermore, in-person household surveys are more difficult because rural regions are less densely populated, which means prohibitively long travel distances to obtain sufficient population samples [13].

To address these unique challenges of rural health surveillance, we applied an address-based sampling frame to phone and mailed surveys to capture a geographically precise population sample [14]. We also analyzed the sociodemographic and geographic reach of in-person surveys based on convenience sampling, which is one strategy already employed in Sullivan County. Our goal was to find a cost-effective method of surveying a geographically specific population sample. Doing so would enable sub-county estimates of disease burden that would help target health interventions to local areas in rural America that need them most [15].

## Methods

### Study design overview

We administered a brief health survey by mail, by phone, and in-person in New York’s rural Sullivan County. All surveys were performed contiguously in May 2017. We compared response rates, estimated costs, respondent demographics, and prevalence estimates among the three modalities. We also compared how response rates were affected by $10 versus $20 incentives for participation.

### Study population

The study population included adult residents of Sullivan County aged 18 years or older in ZIP code 12754, which is located in the town of Liberty, New York. Based on American Community Survey (ACS) estimates from 2013 to 2017, the ZCTA of 12754 has an adult population of 5375. We selected a single ZIP code to identify the most cost-effective method of delivering geographically targeted health surveys before distributing surveys to other ZIP codes throughout the county. Through each survey modality, we attempted to contact 200 adults for a total sample of 600 (see Additional file 1: Appendix for survey questions consistently used in all three modalities).

### Mailed surveys

We obtained address point and parcel data for all mailing addresses in ZIP code 12754 from the New York State GIS Clearinghouse (www.gis.ny.gov, May 2017). This data source was selected because it also listed property classes and land use data for all addresses. We included any residential listing that was not marked as seasonal or vacant housing. We also included commercial addresses listed as apartments. We randomly selected 200 address points for the mailed survey. Each mailing included one survey per address and a return envelope addressed to the Sullivan County Public Health Services with postage attached. The incentive for participation was randomly assigned as a $10 or $20 gift card received by mail. Respondents were given 3 weeks to return surveys given that there is a substantial influx of seasonal non-residents who arrive in Sullivan County over the summer.

### Phone surveys

We selected another non-overlapping random sample of address points in the same manner described above for the phone survey. To obtain phone numbers associated with each address, we used the reverse address lookup directory in the premium version of Whitepages.com. This site provides online directory services and is the largest available database of contact information for U.S. residents. It claims to have phone and address records for over 90% of Americans [16]. As opposed to random-digit-dialing, this approach allowed us to geographically target phone numbers in a specific county and ZIP code.

Individuals in the database are listed in chronologic order with the most recent resident listed first. Therefore, we chose the first person associated with each address who was not noted to be deceased. We also required that the main or primary phone number was currently registered to a Sullivan County resident in ZIP code 12754. Most of these primary phone numbers were landlines, which we restricted to the 845 area code that fully covers ZIP code 12754. When the primary number was a mobile phone based on data from the directory, it also had to be currently registered to a Sullivan County resident in ZIP code 12754. We also scanned secondary phone numbers for additional mobile phone numbers that met these criteria. A total of 200 addresses with valid associated phone numbers meeting above requirements were selected for the phone survey. These addresses were randomly assigned to have a $10 versus $20 gift card received by mail as an incentive for participation.

Three rounds of calls were made to increase the likelihood of reaching a resident at each address. Calls were made on weekdays between 6:00 p.m. and 8:30 p.m., which were times in the evening when our research staff were available. In the first round, calls were made without leaving a voicemail. In the second round, a voicemail was left to see if any return calls were made. In the third round, calls were also made without leaving a voicemail. To maximize the likelihood that an individual would pick up the call, we used Google Voice so that the area code listed on caller identification systems would show an 845 area code in the city of Monticello, New York (county seat of Sullivan County).

### In-person surveys

For in-person surveys, we approached 200 individuals at a strategically central location with a large amount of diverse foot traffic, which is one type of health surveillance strategy already employed in Sullivan County. We chose this convenience sampling design because an address-based, in-person household survey would not be cost-effective in the county’s rural and mountainous regions. We selected one of the two large grocery stores in ZIP code 12754 as the optimal recruitment site. The one chosen was larger and had more customers. Permission was granted to enroll survey participants in the store. At the entrance of the store, we approached the first 200 customers and invited them to complete the brief health survey for a store gift card. In-person surveys were performed on 2 weekdays between 8:00 a.m. and 8:00 p.m., which were times when staff from the Sullivan County Public Health Services were available. As with the mailed and phone surveys, individuals were randomly assigned a $10 versus $20 gift card in-person as an incentive for participation.

### Study outcomes

Our primary outcome was the response rate (AAPOR RR2) as the measure of effectiveness for each modality used to deploy the survey. We also analyzed whether response rates differed between the $10 versus $20 incentives. Using standardized AAPOR (American Association for Public Opinion Research) definitions, we also reported contact (CON1), cooperation (COOP2), and eligibility (ELR) rates (see Additional file 1 for equations). During the study, we tracked the total person-hours required to deliver the health survey for each modality and other costs. Secondary outcomes included respondent demographics (age, gender, race, and ethnicity) compared to Census estimates for ZCTA (ZIP code tabulation area) 12754 from the ACS in 2013–2017.

Other secondary outcomes were prevalence estimates obtained for key health conditions. Each survey respondent was asked whether they had ever been told by a doctor, nurse, or health professional that they had hypertension, hyperlipidemia, diabetes, or asthma. Questions were also asked to assess body-mass-index and smoking status. All questions were derived from the BRFSS. For each health condition, unadjusted crude prevalence was calculated in addition to age-adjusted prevalence using the direct method described by the CDC [17].

### Statistical analysis

For our primary and secondary outcomes, 95% confidence intervals were calculated using the binominal exact method. Rates and proportions were compared using Fisher’s exact Chi-squared tests to identify statistically significant differences. To estimate costs, total person-hours were multiplied by a labor cost per hour and added to other costs required to deploy surveys through each modality. For prevalence estimates, we calculated 95% confidence intervals for unadjusted and age-adjusted rates across three age strata (18 to 44, 45 to 64, and 65 and older).

Statistical analyses were performed using Stata 14.2 (StataCorp: College Station, TX, 2015). Mapping was performed using ArcGIS Desktop 10.3.1 (ESRI: Redlands, CA, 2015).

## Results

### Mailed surveys

Out of 200 mailed surveys, 48 were returned to sender, resulting in a contact rate of 76.0%. When reviewed, 22 of the 48 returned were vacant addresses. The rest were undeliverable, unknown, or incorrect addresses. Of the 152 surveys successfully delivered to eligible addresses in ZIP code 12754, the cooperation rate was 31.6%. Of completed surveys, 81.3% were returned the first week, 14.6% the second week, and 4.1% the third week. The overall response rate for mailed surveys was 24.0%. The $20 incentive had a 27.0% response rate compared to 21.0% for the $10 incentive, but this difference was not statistically significant (binomial exact, p = 0.18) (Table 1 and Additional file 1: Figure S1).

**Table 1:** Comparison of survey metrics by modality and incentive

Survey metrics	Mailed survey	Phone survey	In-person survey
Contact rate			
Overall	76.0% (69.5–81.7%)	33.2% (26.6–40.2%)	100% (Not Applicable)
$10 Incentive	73.0% (63.2–81.4%)	27.6% (19.0–37.5%)	100% (Not Applicable)
$20 Incentive	76.0% (69.7–86.5%)	38.8% (29.1–49.2%)	100% (Not Applicable)
Cooperation rate			
Overall	31.6% (24.3–39.6%)	24.6% (14.8–36.9%)	71.4% (61.4–80.1%)
$10 Incentive	27.6% (18.0–39.1%)	33.3% (16.5–54.0%)	57.8% (42.2–72.3%)
$20 Incentive	35.5% (24.9–47.3%)	18.4% (7.7–34.3%)	83.0% (70.2–91.9%)
Eligibility rate			
Overall	100% (Not Applicable)	80.0% (56.3–94.3%)	40.7% (33.3–48.4%)
$10 Incentive	100% (Not Applicable)	81.8% (48.2–97.7%)	32.1% (22.2–43.4%)
$20 Incentive	100% (Not Applicable)	77.8% (40.0–97.2%)	48.4% (37.7–59.1%)
Response rate			
Overall	24.0% (18.3–30.5%)	8.2% (4.7–12.9%)	71.4% (61.4–80.1%)
$10 Incentive	21.0% (13.5–30.3%)	9.2% (4.3–16.7%)	57.8% (42.2–72.3%)
$20 Incentive	27.0% (18.6–36.8%)	7.1% (2.9–14.2%)	83.0% (70.2–91.9%)
Eligible survey responses			
Total number	48 of 200	16 of 200	70 of 200

Survey metrics based on standardized AAPOR definitions: contact rate (CON1), cooperation rate (COOP2), eligibility rate (ELG), and response rate (RR2)

95% confidence intervals in parentheses

### Phone surveys

To obtain 200 primary phone numbers with our reverse address lookup method, we sampled 248 random addresses of which 19.4% did not result in a landline with an 845 area code or a mobile phone registered to a living person at that address. Of the 200 primary phone numbers obtained, 92.5% were landlines and 7.5% were mobile phones. After three rounds of calls, our contact rate was 33.2%. Of these contacts, 60.9% were reached in the first round, 27.5% in the second round, and 11.6% in the third round.

Among those contacted by primary phone number, 80.0% were eligible as they lived in the geographically targeted ZIP code, and the cooperation rate was 24.6%. The cooperation rate among those offered the $10 incentive was actually higher than those offered the $20 incentive (33.3% versus 18.4% respectively). However, this difference was not statistically significant (binomial exact, p = 0.13). The overall response rate for phone surveys was 8.2% with no difference between response rates at the $10 versus $20 incentive (Table 1).

Only one person responded to 60 voicemails that we left in the second round of phone calls, and that person declined to participate. In addition, we also found 39 mobile phones listed among secondary phone numbers. Using these secondary numbers, we reached 9 additional people. However, 33.3% verbally refused, 22.2% hung up, 44.5% were ineligible based on their ZIP code, and none actually completed the survey. Because of the lack of success with these secondary phone numbers and the high rate of ineligibility, we reported results for phone surveys only using primary phone numbers.

### In-person surveys

We approached 200 individuals for in-person surveys at one of the two large grocery stores in ZIP code 12754. Since surveys were in-person, the contact rate was 100% and reported cooperation rates were effectively the same as response rates because there were no non-contacts. However, among the 172 individuals who agreed to participate, only 40.7% were eligible since many people approached did not live in the geographically targeted ZIP code.

Among respondents who agreed to participate but were not eligible, only two individuals did not live in Sullivan County. The rest of the non-eligible respondents lived in 27 other neighboring ZIP codes within Sullivan County. Three of these neighboring ZIP codes had 7 to 11 respondents, twelve had 3 to 5 respondents, and twelve others had 1 to 2 respondents (see Additional file 1: Figure S4). For in-person surveys, the overall response rate was 71.4% with a significantly lower response rate for the $10 versus $20 incentive (57.8% versus 83.0% respectively, binomial exact, p = 0.01) (Table 1)

### Comparison of estimated costs

Effort required to mail health surveys to 200 addresses was 10 person-hours, which included printing surveys and envelopes, applying postage and mailing labels, and enclosing the surveys by hand. For each mailed survey, we used a first-class 49 cent stamp to mail the survey and a first-class 49 cent stamp on return envelopes. For completed mailed or phone surveys, we also used a first class 49 cent stamp to mail gift card incentives. Each envelope cost 2 cents and each survey cost 5 cents to print. Three rounds of calls to the 200 primary phone numbers required 500 total calls and 18 person-hours or approximately 2 min per attempted call. Approaching 200 individuals in-person required 9 person-hours.

Assuming labor costs of $15 per hour and including incentive costs of $10, mailed surveys were $18.09 per completed survey, phone surveys were 55% higher at $28.01 per completed survey, and convenience in-person surveys were 33% lower at $12.07 per completed survey. At labor costs of $10 per hour, phone surveys would have been 31% more expensive and in-person surveys 12% less expensive. In order to obtain responses for 6000 adults (just over 10% of the adult population in Sullivan County), it would cost approximately $100,000 by mail, $170,000 by phone, and $70,000 in-person. However, the geographic distribution of these samples may differ substantially between methods.

### Demographic comparisons

There was a statistically higher proportion of adults aged 65 and older who responded to mailed surveys (39.6%) compared to Census estimates (20.3%). There was also a substantially lower proportion of adults aged 18 to 44 who responded to the phone survey (18.8%) compared to Census estimates (42.5%). However, this large discrepancy was not statistically significant due to the small number of responses obtained by phone. Finally, all survey modalities had a substantially lower proportion of Hispanic respondents compared to Census estimates (Table 2 and Additional file 1: Figure S2).

**Table 2:** Comparison of demographics of survey respondents by survey modality

Demographics	Mailed survey	Phone survey	In-person survey	Census estimates
Adults	48 responses	16 responses	70 responses	5375 estimated
Age strata				
18–44 years	31.2% (18.7–46.3%)	18.8% (4.0–45.6%)	34.9% (23.5–47.6%)	42.5%
45–64 years	29.2% (17.0–44.1%)	56.3% (29.9–80.2%)	43.9% (31.7–56.7%)	37.2%
65 and older	39.6% (25.8–54.7%)	25.0% (7.3–52.4%)	21.2% (12.1–33.0%)	20.3%
Gender				
Male	35.4% (22.2–50.5%)	31.2% (11.0–58.7%)	23.9% (14.3–35.7%)	48.6%
Female	64.6% (49.5–77.8%)	68.8% (41.3–89.0%)	76.2% (64.1–85.7%)	51.4%
Race/ethnicity				
White	85.4% (72.2–93.9%)	68.8% (41.3–89.0%)	78.3% (66.7–87.3%)	71.0%
Black	4.2% (0.5–14.3%)	6.2% (0.2–30.2%)	8.7% (3.3–18.0%)	6.3%
Hispanic	8.3% (2.3–20.0%)	18.8% (4.0–45.6%)	7.2% (2.4–16.1%)	20.7%
Asian	0.0% (0.0–7.4%)	0.0% (0.0–20.6%)	0.0% (0.0–5.2%)	0.9%
Other	2.1% (0.1–11.1%)	6.2% (0.2–30.2%)	5.8% (1.6–14.2%)	1.1%

95% confidence intervals in parentheses

### Prevalence estimates

Among the three survey modalities, unadjusted prevalence estimates of health conditions were relatively similar in magnitude with the exception that the phone survey estimates often did not fall within the 95% confidence interval for estimates for mailed and in-person surveys. At times, age-adjustment worsened the discrepancy between estimates obtained from phone surveys and those obtained from mailed and in-person surveys. This result was mostly likely due to the small sample size and skewed age distribution obtained by phone (Table 3 and Additional file 1: Figure S3).

**Table 3:** Comparison of health outcomes prevalence estimates by survey modality

Health outcomes	Mailed survey	Phone survey	In-person survey
Adults	48 responses	16 responses	70 responses
Unadjusted prevalence			
Hypertension	43.8% (29.2–58.3%)	50.0% (22.5–77.5%)	35.7% (24.2–47.2%)
Hyperlipidemia	41.7% (27.2–56.1%)	25.0% (1.2–48.8%)	37.1% (25.5–48.7%)
Diabetes	10.4% (1.5–19.4%)	18.8% (0.0–40.2%)	18.6% (9.2–27.9%)
Ever asthma	12.5% (2.8–22.2%)	25.0% (1.2–48.8%)	20.0% (10.4–29.6%)
Current asthma	6.3% (0.0–13.4%)	12.5% (0.0–30.7%)	5.7% (0.1–11.3%)
Ever smoking	39.6% (25.2–53.9%)	68.8% (43.2–94.3%)	38.6% (26.9–50.3%)
Current smoking	12.5% (2.8–22.2%)	31.3% (5.7–56.8%)	21.4% (11.6–31.3%)
Obesity	39.6% (25.2–53.9%)	37.5% (10.9–64.1%)	54.3% (42.3–66.2%)
Age-adjusted prevalence			
Hypertension	31.5% (18.6–44.5%)	59.0% (26.0–92.1%)	32.4% (20.8–44.1%)
Hyperlipidemia	34.8% (19.6–50.1%)	16.1% (5.4–26.7%)	33.2% (22.1–44.3%)
Diabetes	8.2% (1.1–15.3%)	11.8% (0.2–23.5%)	12.9% (6.5–19.3%)
Ever asthma	11.7% (1.8–21.7%)	15.2% (2.1–28.2%)	23.9% (12.3–35.5%)
Current asthma	5.2% (0.0–11.1%)	6.6% (0.0–15.8%)	6.9% (0.0–13.7%)
Ever smoking	40.3% (24.0–56.7%)	67.2% (32.8–100%)	40.1% (27.2–53.0%)
Current smoking	14.4% (2.7–26.1%)	16.6% (5.7–27.5%)	24.8% (13.2–36.5%)
Obesity	41.7% (25.3–58.1%)	49.6% (15.2–84.0%)	57.3% (44.3–70.4%)

95% confidence intervals in parentheses

## Discussion

For a rural public health department, cost-effective health surveillance is needed to identify how disease burden varies at a sub-county level [18]. In Sullivan County, there are 50 ZIP codes that are not post-office boxes (38 fully within, 4 mostly within, and 8 with less than half of their area within the county). In this study, we geographically targeted one ZIP code to determine the most cost-effective strategy prior to performing a county-wide survey of all ZIP codes. By knowing how the prevalence of key health conditions varies among these ZIP codes, it would be possible to identify critical hotspots of disease, which can help rural public health departments focus their limited resources in areas with a higher burden of poor health [19].

For mailed surveys, almost a quarter were returned to sender. A few of these were due to address errors, which may have been avoided by using an address verification service [20]. However, many were vacant despite our having excluded vacant residences based on publicly available land use records. Even so, the mailed survey response rate was 24%, three times higher than the phone survey. For context, most surveys mailed once generally have response rates of 10% to 20% [21]. Rates may be improved by sending follow-up mailings, but these will increase costs and some studies suggest the non-response bias in a single mailing does not differ substantially from multiple mailings [22]. In addition, ineligibility (not living in the targeted ZIP code or Sullivan County) was not a factor as surveys were directly mailed to addresses within the geographically targeted ZIP code. This design would make it possible to ensure that enough respondents are obtained per ZIP code in a county-wide health survey using sampling quotas [23].

For phone surveys, we found no apparent benefit of leaving voicemails or using secondary phone numbers from the reverse address lookup [24]. The reverse address lookup method was also not successful for nearly 20% of addresses queried, which may introduce another source of sample bias by missing individuals whose information is not publicly available [25]. The overall response rate of phone surveys was 8%, which is consistent with recent studies of telephone-based surveys [26]. The Pew Research Center estimates that 20 years ago phone survey response rates were 36%, 10 years ago they were 21%, and now they are down to 9% [27]. This low response rate meant that over an hour of effort was required to obtain each completed survey. For a rural public health department with limited resources, phone surveys currently require too much effort to perform.

**Fig. 1:**
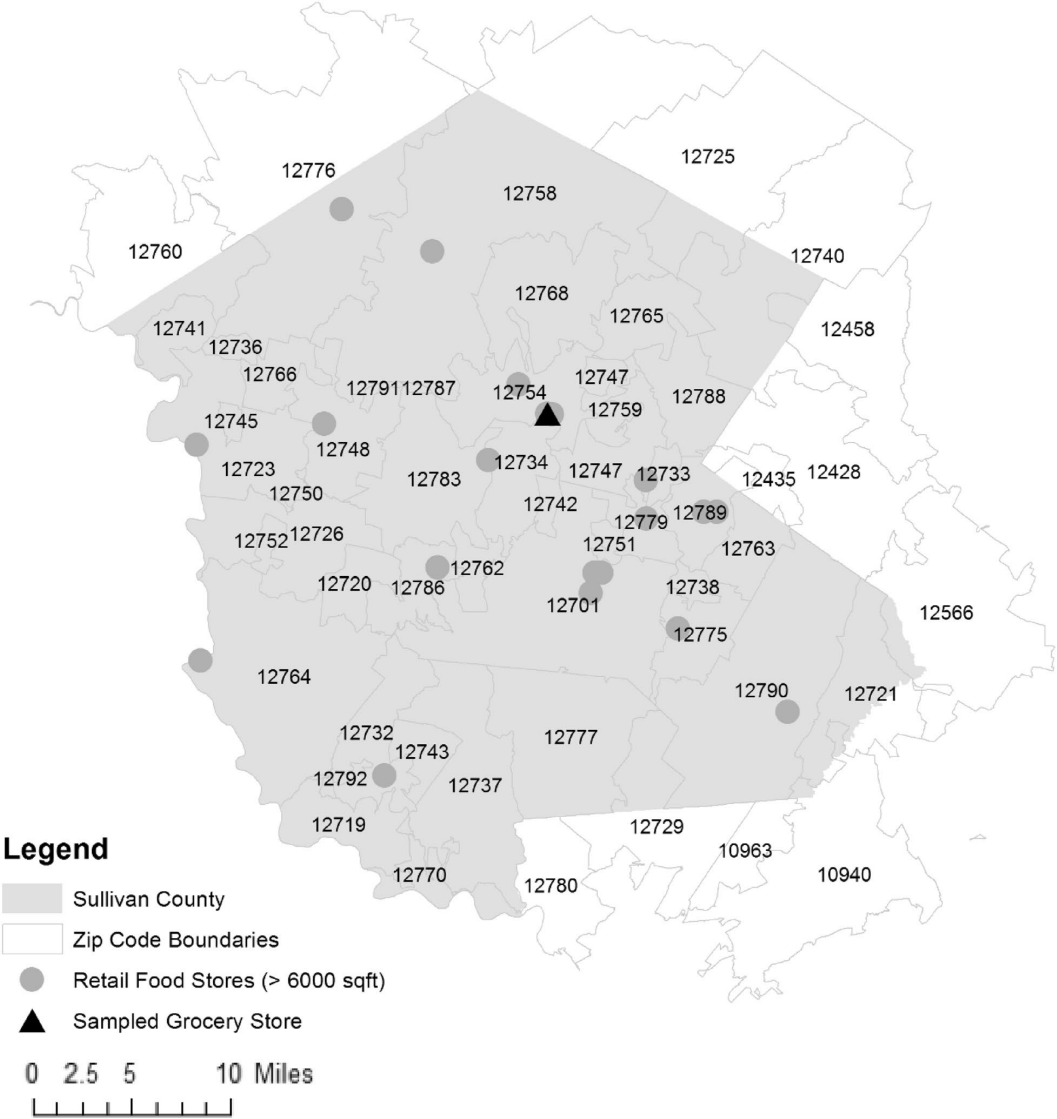
Sullivan County ZIP codes and retail food stores. ZIP codes fully and partially within Sullivan County New York. Retail food stores greater than 6000 square feet mapped based on data from the New York State Department of Agriculture and Markets

The type of in-person survey performed in our study had a much higher response rate and appeared less costly to perform. However, there was no clear sampling frame in this approach, which was a convenience sample of a single site [28]. There are 20 other large retail food stores in Sullivan County, but not in every ZIP code (Fig. 1 based on retail food store data from the New York State Department of Agriculture and Markets) [29]. Notably, some of these stores serve a specific customer base and others are not open year-round and only cater to seasonal visitors [30]. Some customers willing to complete in-person surveys at the sampled grocery store lived in neighboring ZIP codes without a grocery store. However, their number were so few that the time and effort to obtain an adequate sample for these neighboring ZIP codes would dramatically increase the costs of this approach. For example, there were only 7 potential survey participants over 9 h from neighboring 12759, an adjacent ZIP code without a grocery store. Thus, it would have taken ten times longer to obtain the same sample size of 70 that was obtained for originally targeted ZIP code 12754.

Among the survey modalities, the in-person survey was the only one with a statistically significant difference in cooperation and response rates that favored the $20 over $10 incentive. It was surprising was that the cooperation rate for the $20 incentive was lower than for the $10 incentive in the phone survey (18% versus 33% respectively). This result may have been by chance since it was not statistically significant, and may also have been due to the small number of responses obtained, especially as the observed magnitude of difference was substantial. One potential explanation for the result, if true, might be that a higher incentive increased perceptions that the survey was a scam. Without a definitive way for the public to confirm phone surveys, many national organizations regularly warn consumers against fraud telephone calls [31].

Notably, all survey modalities had a lower proportion of Hispanic respondents than Census population estimates, which is consistent with the literature on survey participation [32]. For in-person surveys, approaching customers at the other grocery store in the ZIP code where there are more Hispanic customers might have helped. Also, offering mail and phone surveys in Spanish would increase participation among Hispanic residents. Consistent with prior literature, mailed surveys had a higher proportion of adults older than 65, and phone surveys had a lower proportion of adults younger than 45 agree to participate [33]. Some of these biases can be partially corrected by age-adjusting results of the surveys. As for prevalence estimates, we found unadjusted and age-adjusted rates were generally similar for queried health conditions, except for some phone survey results, which likely did not have enough respondents to provide accurate estimates.

Other limitations of this study included that the design only focused on a single rural ZIP code. The reason for this study design was to determine the optimal approach for rural health surveillance prior to deploying a larger county-wide survey. In addition, the convenience sample of the in-person survey may present issues of dependence, therefore, inference may be limited in comparisons with this approach. Furthermore, to obtain our samples, phone and in-person studies were only performed on weekdays and at times when staff were available. For higher reliability in larger scale surveys, it would be important to perform sampling during weekends and other times during the day, which may be more successful in obtaining a representative sample. Our study also did not compare surveys performed without any incentive, which may be a means of reducing survey costs, but may lead to substantially lower response rates.

## Conclusions

In conclusion, current rural health surveillance methods are severely limited by small sample sizes, and there is a strong need to capture a larger proportion of rural residents through more comprehensive public health surveillance. While harnessing healthcare datasets in real time, such as electronic health record networks, holds future promise for rural area health surveillance [9], most rural counties still lack the infrastructure, training, and cross-institution partnerships to do so. Geographically targeted health surveys remain an important surveillance tool to monitor health behaviors and conditions. By providing a cost-effective means of finding hotspots of poor health, we can help match resources and interventions to places where they are needed most in rural America.

Having identified a cost-effective method of deploying a brief health survey in a single ZIP code of New York’s rural Sullivan County, the next step is to perform a county-wide health survey to capture a substantial proportion of the population, but in a geographically specific manner. By performing a county-wide survey of chronic disease prevalence, it would be then possible to evaluate alternative surveillance methods (e.g., using claims data, health information exchanges, or modelling approaches) to estimate rural chronic disease prevalence, which may be easier to sustain. This initial study within a single rural ZIP codes helps to address some of the questions regarding optimal survey design in rural settings.

We believe the results favor a mailed survey and also demonstrates that a higher incentive may not be worth the expense, especially if lower incentives allow for contacting more individuals. There are also ways to automate mailed surveys to reduce the effort required, and strategies to improve mailed survey response rates such as using colored ink and brief surveys have been studied through systematic literature reviews [34]. Furthermore, these results identify the need to address some issues in the representativeness of the population samples obtained whether it be through age-adjustment or amending the surveys to accommodate residents whose primary language is not English.

## Supplementary information

**Additional file 1.** Supplemental Appendix and Figures.

## Data Availability

The datasets used and/or analyzed during the current study (after de-identification and aggregation as needed to prevent identification) are available from the corresponding author on reasonable request

## Abbreviations

CDC: Centers for Disease Control and Prevention
BRFSS: Behavioral Risk Factor Surveillance System
AAPOR: American Association for Public Opinion Research
RR: Response rate
CON: Contact rate
COOP: Cooperation rate
ELR: Eligibility rate
ZCTA: ZIP code tabulation area
