# Supplementary material for: Comparing methods of performing geographically targeted rural health surveillance
Source: Emerg Themes Epidemiol. 2020 Nov 23;17:3. doi: 10.1186/s12982-020-00090-0 (PMC7686693; doi:10.1186/s12982-020-00090-0)
Supplement: Supplementary file 1 — Additional file 1. Supplemental Appendix and Figures. [file 12982_2020_90_MOESM1_ESM.docx]

**Supplemental Appendix: Health Survey Questions**

1. Is your primary home or residence located in Sullivan County?

2. Is your primary home or residence in ZIP code 12754?

3. What is your age?

4. Are you male or female?

5. How long does it take you to get to your post office from home?

6. Is this amount of time above how long it takes by driving or walking?

7. In general, your health is excellent, very good, good, fair, or poor?

Has a doctor, nurse, or other health professional EVER told you that you had:

8. Hypertension or High Blood Pressure

9. High Cholesterol or Hyperlipidemia

10. Diabetes

11. If yes, what kind of Diabetes? Type 1, Type 2, Pregnancy-Related, Not Sure

12. About how tall are you without shoes?

13. About how much do you weigh without shoes?

14. Have you smoked at least 100 cigarettes in your entire life?

15. Do you currently smoke cigarettes every day, some days, or not at all?

16. Has a doctor, nurse of other health professional ever told you that you had asthma?

17. During the last 12 months, have you had an episode of asthma or asthma attack?

18 – 21. [Additional questions were asked about children and their health.]

22. Are you Hispanic, Latino or Spanish in origin?

23. Which of these is your race? White, Black, Asian, or Other?

**Supplemental Appendix: Survey Outcome Definitions**


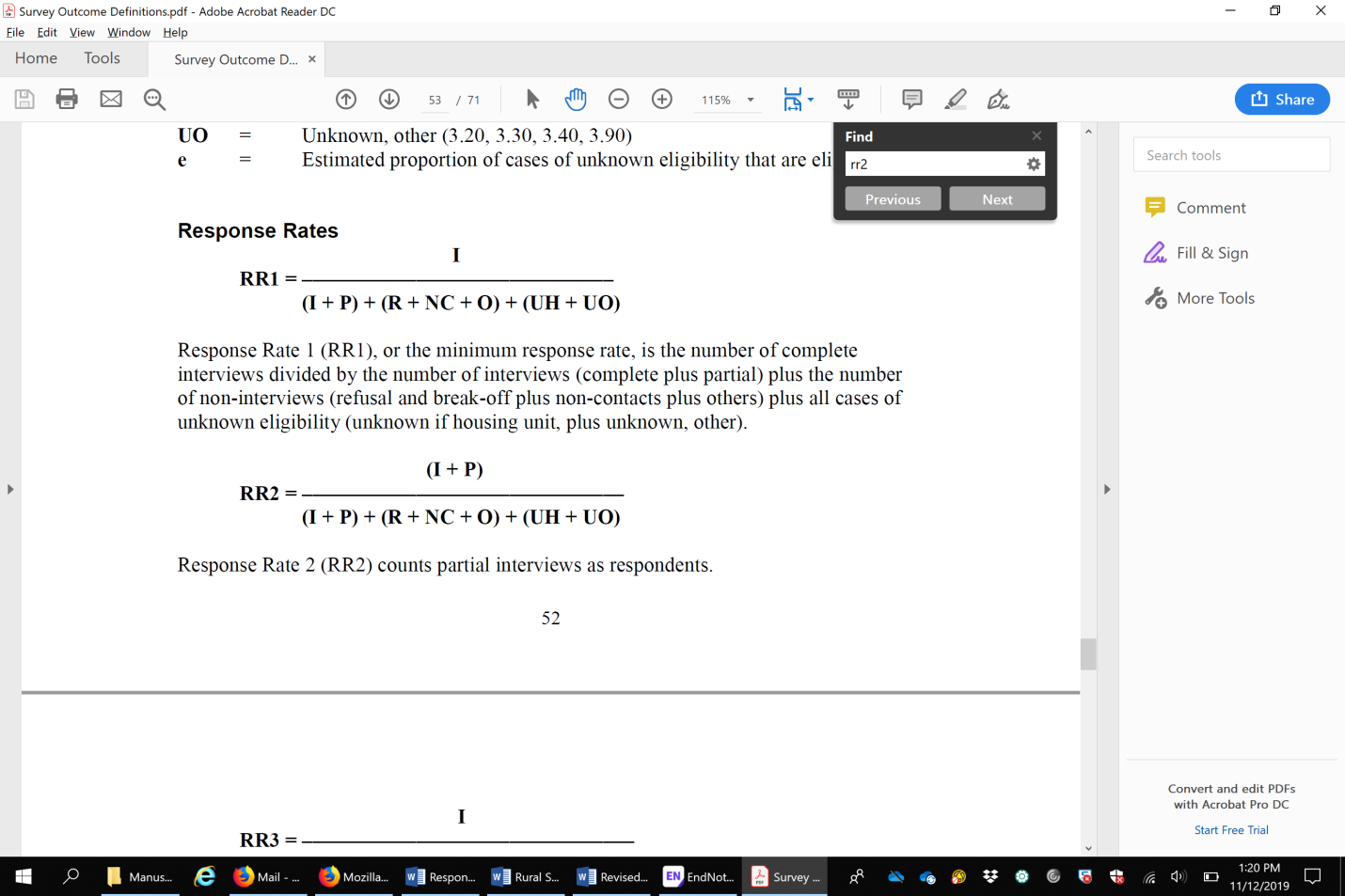


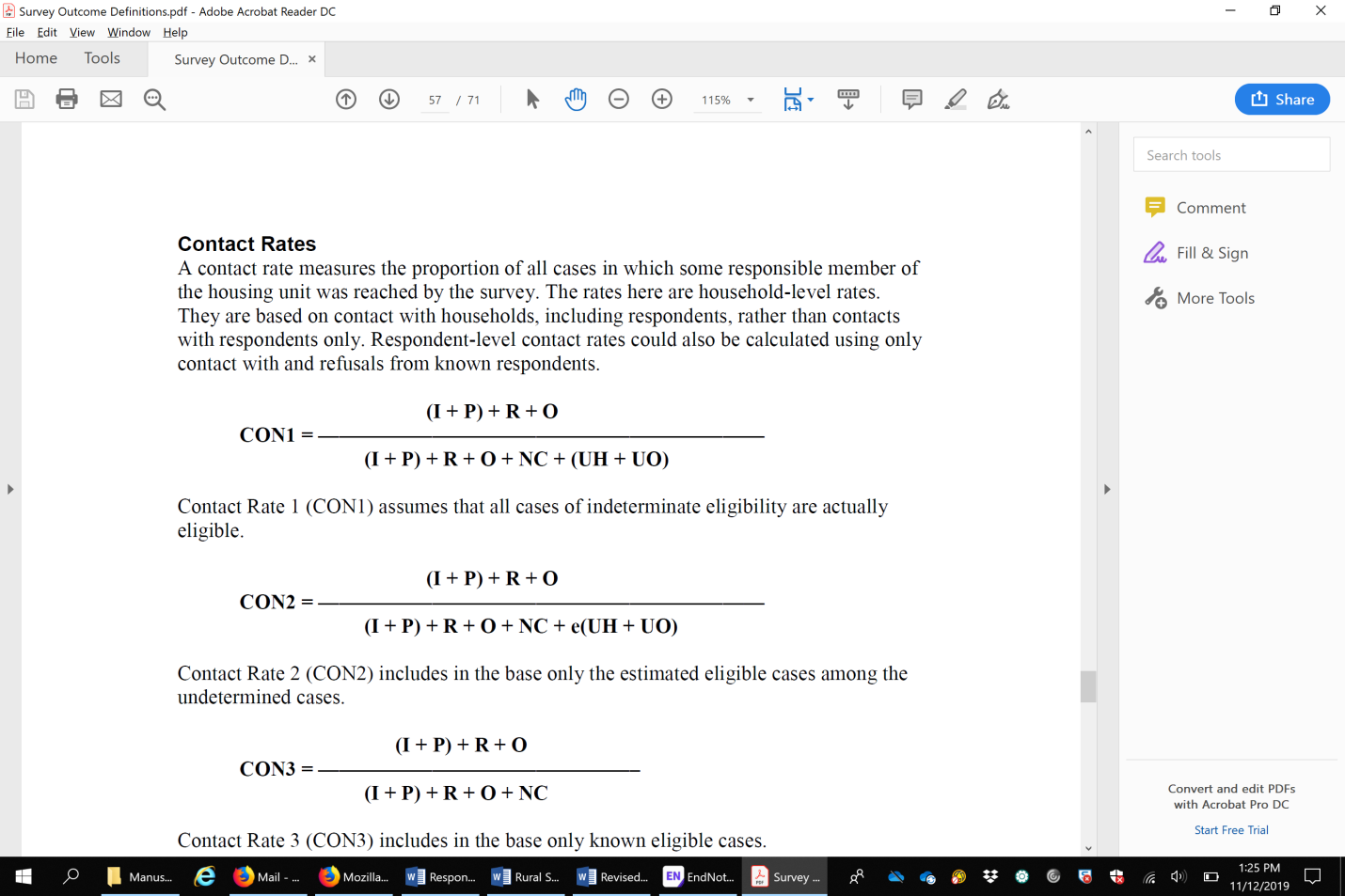


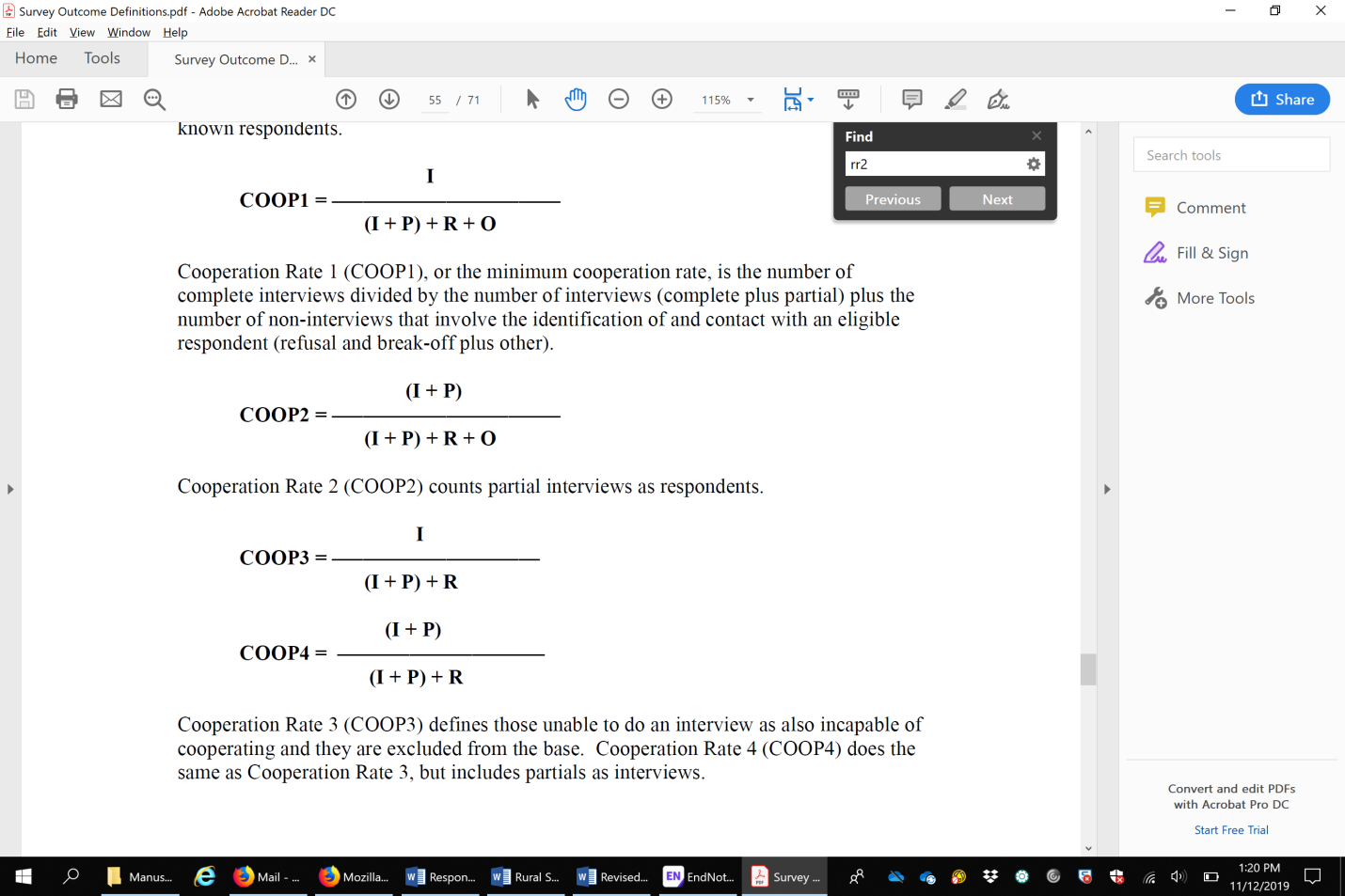


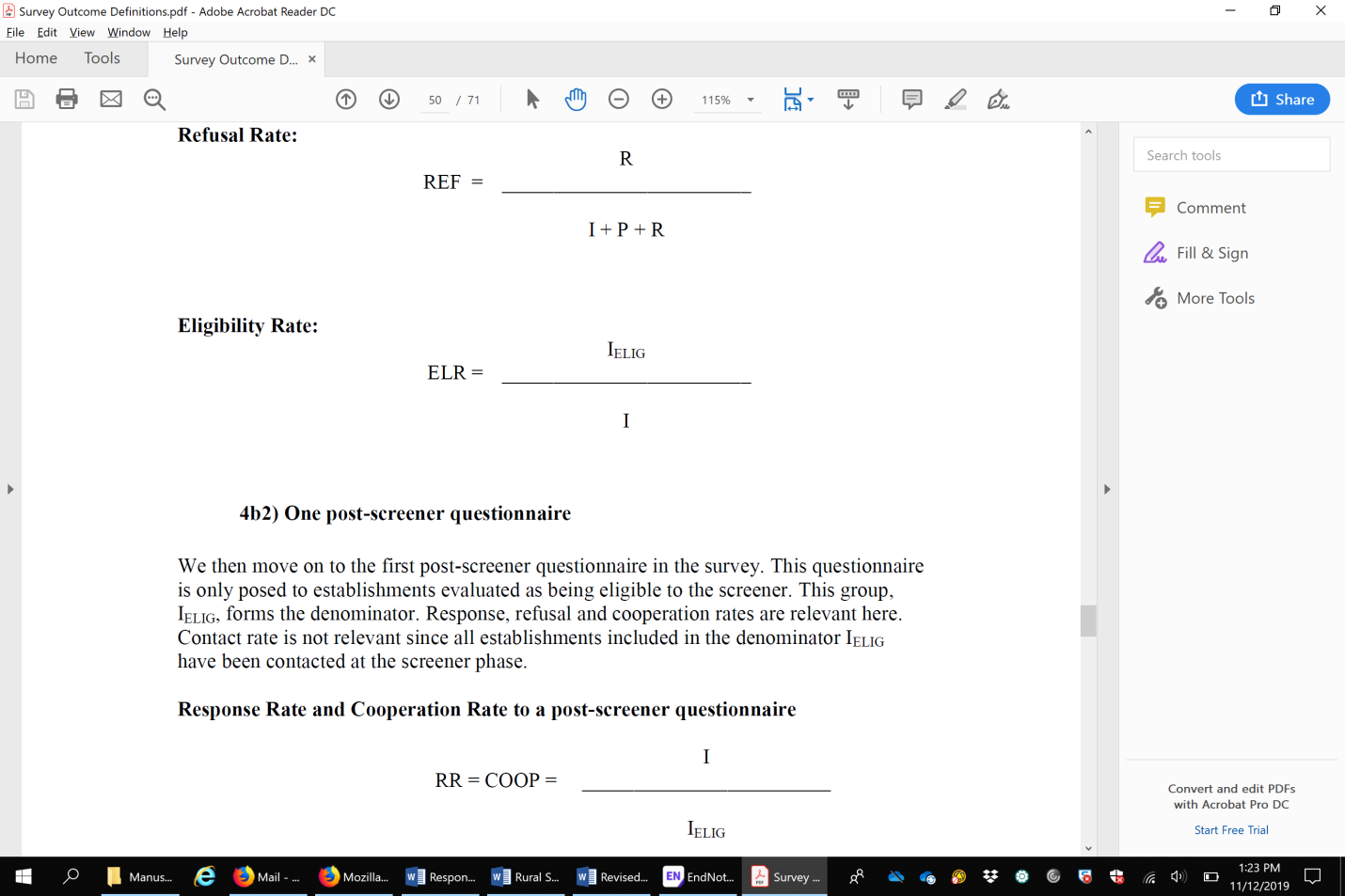


Abbreviations:

RR = Response rate

COOP= Cooperation rate

REF = Refusal rate

CON = Contact rate

I = Complete interview

P = Partial interview

R = Refusal and break-off

NC = Non-contact

O = Other

UH = Unknown if household/occupied

UO = Unknown, other

e = Estimated proportion of cases of unknown eligibility that are eligible

I _ELIG_ = Complete screener questionnaires which are evaluated by the screener instrument as eligible

**Figure S1: Comparison of Survey Metrics by Modality and Incentive**

**Mailed Survey Phone Survey In-Person Survey**

**Figure S2: Comparison of Demographics of Survey Respondents**

**Figure S3a: Comparison of Unadjusted Prevalence Estimates for Health Outcomes**

**
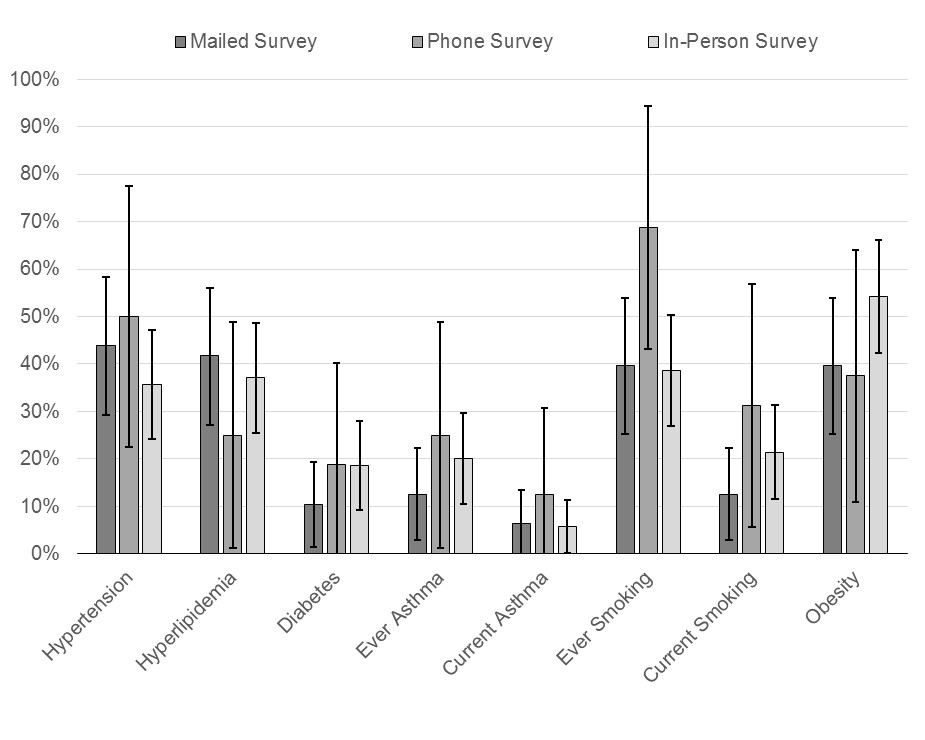
**

**Figure S3b: Comparison of Age-Adjusted Prevalence Estimates for Health Outcomes**

**
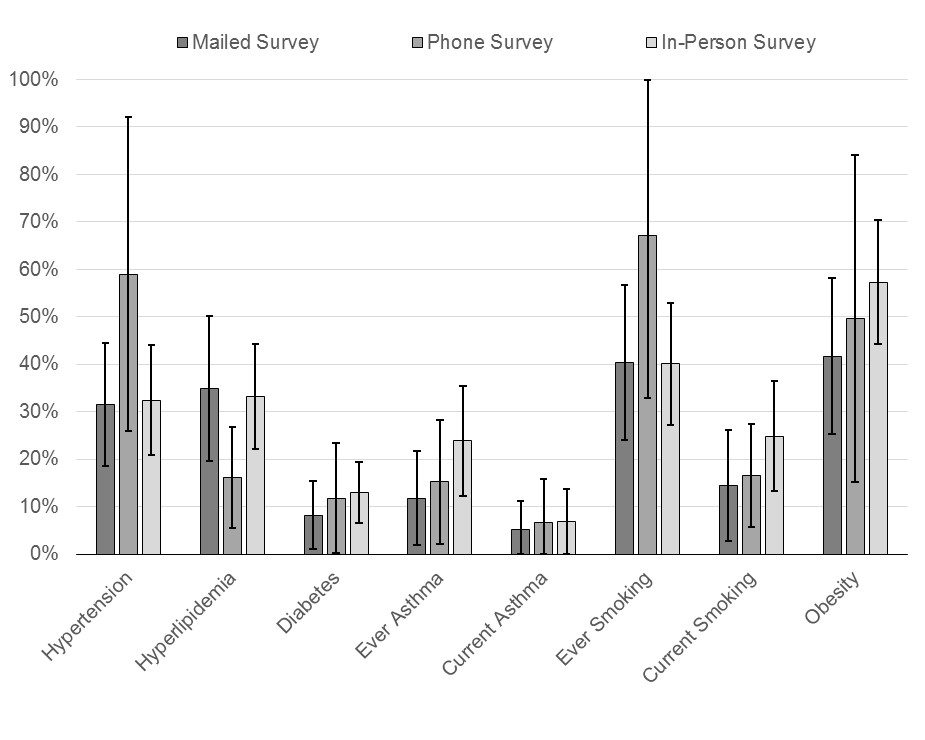
**

**Figure S4: Geographic Distribution of In-Person Survey Respondents Who Agreed to Participate**

**
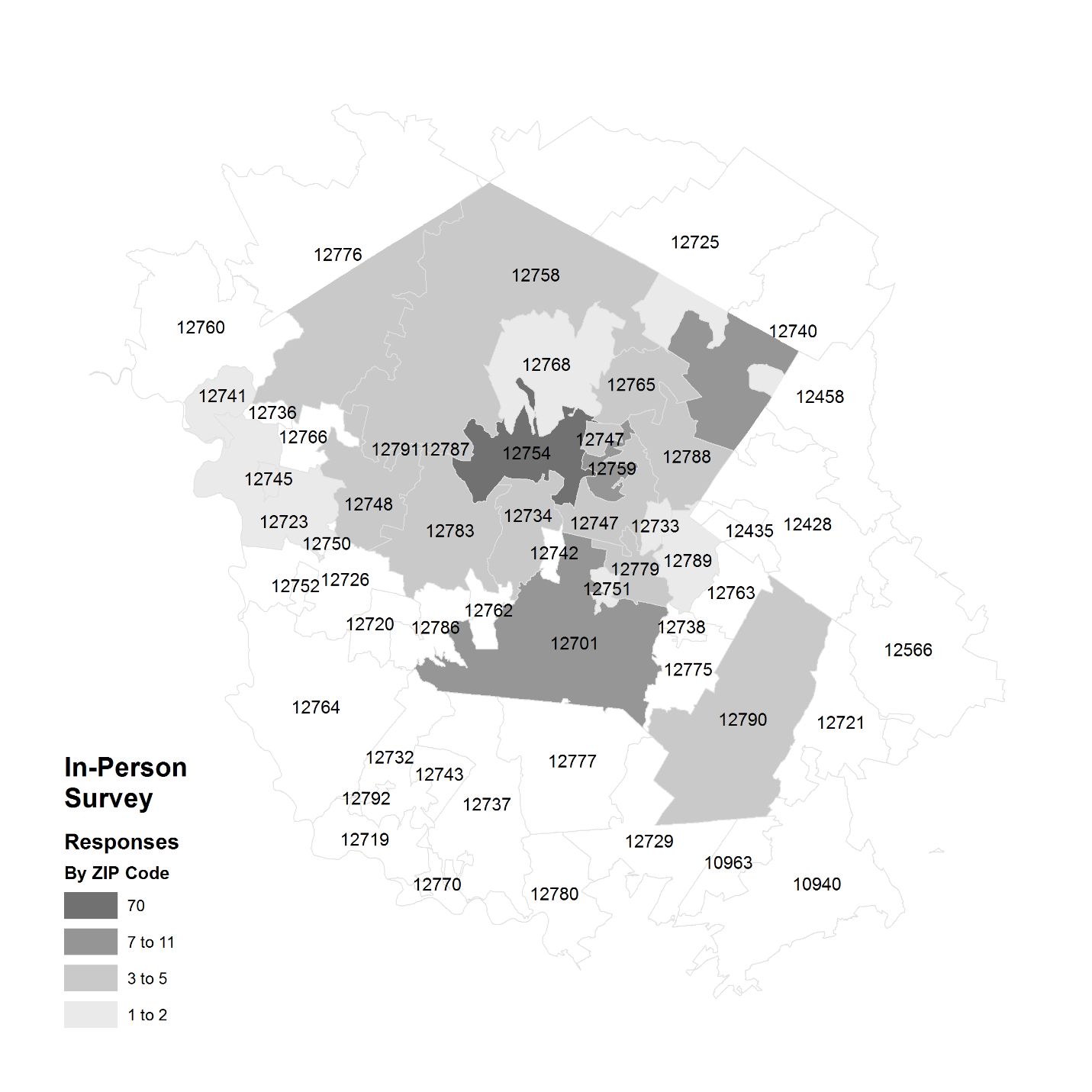
**
